# Fabrication of green anti-microbial and anti-static cement building bricks

**DOI:** 10.1038/s41598-024-56514-3

**Published:** 2024-03-15

**Authors:** Abeer Reffaee, Mona Saied, Shimaa Farag hamieda, Sh. K. Amin

**Affiliations:** 1 Microwave Physics and Dielectrics Department, National Research Centre, Cairo, Egypt; 2 Chemical Engineering and Pilot Plant Department, Engineering and Renewable Energy Research Institute, National Research Centre (NRC), Affiliation ID: 60014618, Giza, Egypt

**Keywords:** Cement, Waste polyvinylchloride (WPVC), Dielectric properties, Antibacterial, Biophysics, Engineering, Materials science, Physics

## Abstract

The design cement mix of grade 350 was created in accordance with Egyptian Standards by partially substituting the fine aggregate with WPVC waste in various weight percentages (10, 20, 30, 40, 50, 75, and 100%). A control mix with 0% replacement was also prepared. The W/C ratio was about 0.5 for all mixes. Compressive, flexure strength, bulk density, and absorption tests were studied. For WPVC replacement, until 30%, compressive strength and flexure strength are acceptable with respect to standerds. Thermal treatment at 200 °C improves the compressive strength, flexure strength and water absorption for 20% WPVC only. The dielectric properties of all cement paste mixes before and after heat treatment, over a frequency range (0.1–10^6^ Hz), were measured as a function of frequency. For dielectric properties and conductivity, an improvement was obtained until 30% WPVC. After this percentage, the dielectric properties and the conductivity got worse. So, cement paste with 30% WPVC as replacement of sand is the optimum ratio with conductivity in range of 10^−12^ S/cm, which is a good choice for antistatic cement paste applications (10^−10^–10^−12^ S/cm). The antimicrobial efficacy of the prepared cement samples of WPVC concentrations (0, 20 and 30) % were studied, the number of grown microbial colonies decreased for all the samples compared to control tap water and decreased by introducing WPVC into the cement paste sample. So, it is recommended to use these samples in places that should be carefully shielded from bacterial infections and static electric charge dangers.

## Introduction

Waste is described as any material that is gaseous, liquid, solid, or a mixture of these media, as well as any unwanted or unnecessary by-product, as well as any emission, discharge, excretion, or residue of a process or treatment^1,2^. When waste is managed correctly, it can be turned into a resource that reduces the need for raw materials, protects the environment and the climate, and encourages sustainable growth. Waste polymers are a serious environmental problem in many nations throughout the world due to increasing production^3^. Waste plastics are the third largest stream of solid waste after food trash and paper waste hence, the majority of municipal and industrial garbage in cities is made up of plastic waste. Even cities with little economic development have begun to generate more plastic garbage as a result of the growth of the plastics industry. On the other hand, post-consumer waste recycling can provide an opportunity to collect and dispose of plastic garbage in the most environmentally responsible manner, and it can be converted into a resource. In most cases, plastic trash recycling could also be economically viable.

Traditional construction materials including concrete, bricks, hollow and solid blocks, pavement blocks, and tiles are made using already-existing natural resources. Because of continuous development and resource depletion, this is damaging the environment^4^. As a result, nature conservation concerns have become increasingly significant in our community in recent years^2,5^. So, the decision-makers in several sectors are increasingly taking environmental challenges more seriously. As a consequence, significant adjustments are being made to our lifestyles and working practices with regard to resource conservation and waste management. Numerous authorities and investigators have now been seeking to obtain the right to reuse the waste in ways that are both environmentally and economically secure^2,6^. One such creative attempt is the use of solid wastes as building materials.

Because of the high demand, low availability of raw materials, and high cost of energy, the cost of construction materials is rising daily. From the standpoint of preserving resources and reducing energy use, using alternative components in building materials is currently a global priority. To supply sustainable and ecologically friendly construction materials, significant research and development efforts are required to uncover new components. The viability of incorporating plastics into concrete mixtures has been studied throughout the past two decades^7,8^. Plastics’ unique properties, such as low density, lightweight, and low cost, make them an excellent choice for use in concrete and cement pastes. By reusing waste and recycled plastic as an addition to the concrete mix and observing the behavior of the resulting concrete, researchers have recently investigated the possibility of waste and recycled plastic as an environmentally beneficial construction material^9^. According to a recent study, artificial plastic aggregate can be utilized as a 25% replacement for natural aggregates, resulting in concrete that is lighter in weight and more thermal insulation while still keeping the necessary strength and ductility for non-structural applications^3,10^. Rabin et al. demonstrated that using recycled PP fibre to reinforce concrete pathways is more environmentally friendly than using SRM or virgin PP fiber when considering environmental sustainability^11^. Additionally, various reactive material treatments of polyethylene provide improvements in the density, compressive strength, and water absorption of concrete that contains treated polyethylene as aggregate^12^.

Poly (vinyl chloride), also known as PVC, is the most important of the vinyl thermoplastics in terms of volume of manufacturing and scope of application, with commercial goods ranging from extremely hard to extremely flexible. PVC is a universal polymer due to its low cost and good performance, as well as the large range of products that may be created from various processing conditions and procedures. As a result, PVC wastes are rapidly increasing, and the quantity of used PVC items entering the waste stream is steadily increasing as a higher number of such PVC products are produced^2,4^. Because of its high chlorine concentration (56.7%), PVC is not easily burned. Moreover, when PVC degrades thermally, harmful byproducts such dioxins and hydrogen chloride may be produced. Thus, the optimum method of recycling PVC is mechanical^13^.

The completion of further qualities, among which it is currently able to specify the water vapour permeability and vapour sorption isotherm, resistance to fire, and resistance to rodents, pestes, etc., is necessary for future work and before employing them within structures. Moreover, research will need to be done on the antibacterial qualities of these novel materials (i.e., their resistance to fungus). In some hostile environments, such as sewer systems, maritime engineering, structures exposed to high humidity, and the like, concrete constructions are easily damaged by microbial adhesion, colonization, and eventually degeneration^14,15^. As sulfur-oxidizing bacteria (SOB) of the genus Thioalbacillus convert hydrogen sulphide (H_2_S) gas into sulfuric acid, microbially caused corrosion is still frequently referred to as a sulphide (H_2_S) gas issue^16–18^, and certain fungi also engage in this activity^16^. Concrete’s internal structure will be destroyed, its mechanical characteristics will deteriorate, and its durability will decrease, driving up the cost of replacement and even rehabilitation^19,20^. Thus, the creation of antimicrobial concrete has become both incredibly important and necessary for smart and long-lasting infrastructures. According to^21^, concrete with zeolite holding silver displayed antibacterial properties.

Finally, the relative permittivity, also known as the dielectric constant is a property of a material related to the density of the electric dipoles. The dielectric constant has been established in order to comprehend the dielectric behaviours of cement-based materials since electric dipoles are present in cement as a result of ionic bonding and moisture. These behaviours looked at how moisture^22,23^ and various admixtures affected the mixture. An AC electric field must be applied to the material under investigation in order to estimate the dielectric constant. In our research, the parallel-plate capacitor used for measurement has the specimen sandwiched between its two electrodes, and an AC electric field is subsequently supplied across the capacitor. This approach is utilised in this study since it enables us to determine the quantitative value of dielectric constant. Finally, our research aimed to produce cement paste having certain specifications in allow for it to be utilized in buildings need to be carefully shielded from bacterial infections and static electric charge dangers.

## Experimental

### Materials and methods

The experiments were done on the produced green cementanous mortars using waste polyvinyl chloride (WPVC) pipes wastes. For the antimicrobial study, we used nutrient agar medium (NA). A general culture medium for less fastidious microorganisms as well as for permanent cultures was prepared from peptone meat extract NaCl and agar of concentrations (0.5, 1.0, 5.0, and 15.0 g/l) and pH 7.4 ± 0. Nutrient broth medium (NB) was prepared from yeast extract, peptone, meat extract, and NaCl with concentrations of 2.0, 5.0, 1.0, and 5.0 g/l, respectively. One set of samples, tap water from NRC, and 100.0 ml sterile conical flasks. Gram-negative bacteria: *Escherichia coli *(*ATCC *25922)*;* Gram-positive bacteria: Staphylococcus *aureus* (ATCC 6538); and pathogenic yeast: *Candida albicans* (ATCC 10231).

The utilization of these wastes by partial replacement of fine aggregates (natural sand) to produce green cementanous mortar, which was applied in the production and manufacture of different sustainable construction materials such as: concrete building bricks, solid non-load-bearing concrete masonry units, cement facing bricks, …. etc.

#### The raw materials

**Figure 1:**
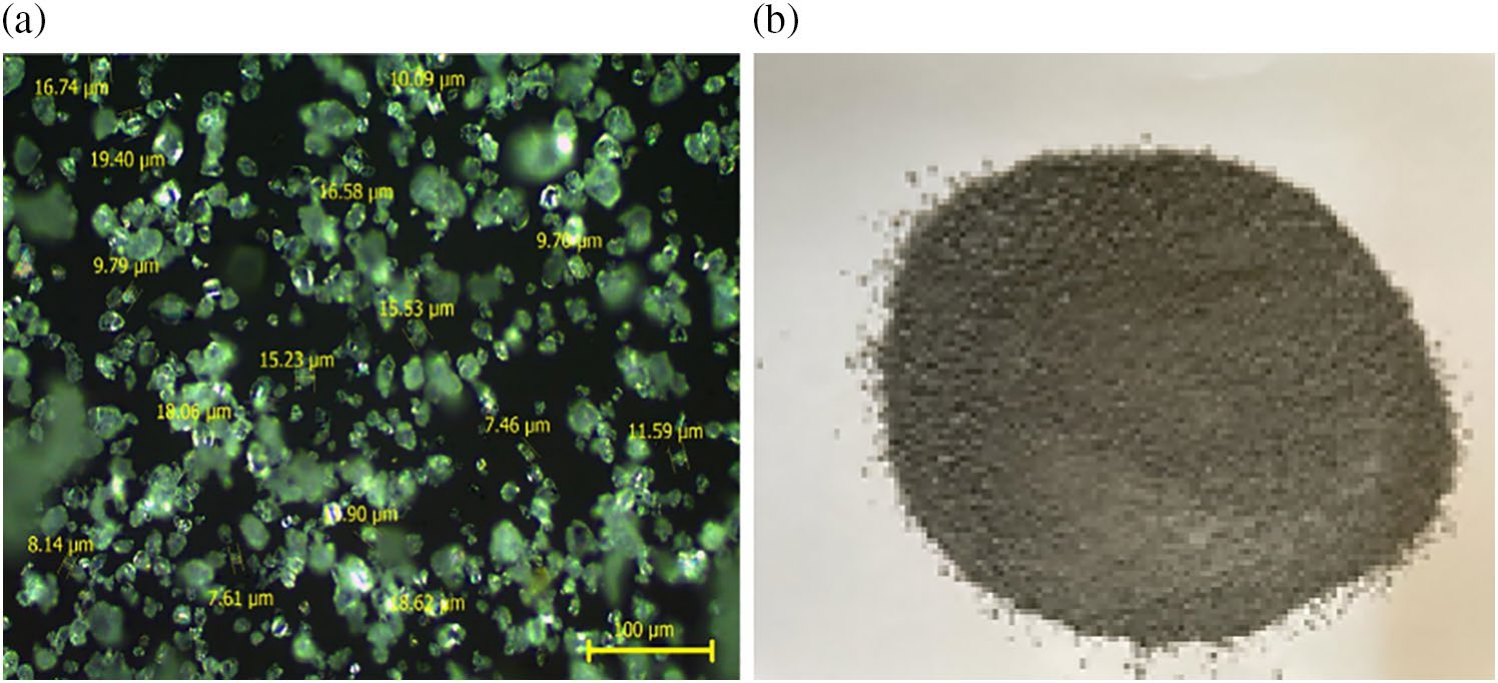
(**a**) POM micrograph and (**b**) an image of waste WPVC powder.

Polyvinyl chloride pipes waste powder (WPVC) was brought from one of the local factories in Fayoum Governorate concerned with plastic recycling. The particle size of WPVC powder was evaluated using Leica DM750P polarizing microscope (POM) with optical lens with a magnification of 20 × (LC Lab NRC), illustrated in Fig. 1a. Figure 1b shows a photograph of WPVC powder.

Ordinary/commercial Portland cement (OPC) [CEM I 42.5 N from Qena Cement Company] was employed in this investigation as a binding material. This kind of cement satisfies the EN 197-1/2011 and ES 4756-1/2013 European and Egyptian cement standards, respectively. The cement had a 3.15 specific gravity and passed through filter number 170 with a 9% fineness. It took 2 h and 3 h and 12 min to reach its initial and final settings, respectively.

Fine aggregate with a maximum size of 4.75 mm that was readily accessible locally was employed (Natural sand from pyramids quarries Giza).

According to Egyptian Standards, a design cement mix of grade 350 was created by substituting a portion of the fine aggregate with WPVC in various weight percentages (10, 20, 30, 40, 50, 75, and 100%). A control mix that included 0% replacement was also created. All of the aforementioned mixes have a W/C ratio of 0.5. All of mixes including the control one, were created to study the impact of fine WPVC partial replacement instead of fine aggregates on its physical–mechanical properties. Compressive, flexure strength, bulk density, and absorption tests were selected for this purpose.

For the compression test, 50 × 50 × 50 mm^3^ cube specimens were cast, and 25 × 30 × 150 mm^3^ prism specimens were produced for the flexure strength test. Following the mixing process, mixtures were poured into moulds and left there for 24 h. The specimens were then cured for 28 days in a curing basin.

Bulk density is widely used in the evaluation and comparison of product quality and as part of the criteria for selection. On the other hand, cold water absorption, which is the relationship of the mass of water absorbed to the mass of dry specimen. These two properties were calculated and reported.

Determination of the breaking load and transverse breaking strength (flexure strength) for the prism specimens of (25 × 30 × 150) mm^3^ after 28 curing days, by applying a force at a specified rate to the centre of the specimen, the point of application being in contact with the proper surface of the specimen. The used apparatus is installed at the Laboratory of the Chemistry Association.

Also, using a universal testing equipment of 1000 KN (Shematzo), which is established at Faculty of Engineering—Cairo University, the compressive strength for cube specimens of (50 × 50 × 50) mm^3^ after 28 curing days was measured.

#### Dielectric measurements

The samples’ conductivity and dielectric characteristics were assessed using a high-resolution broadband impedance analyzer (Schlumberger solartron 1260). The frequency range of the applied AC electric field was 0.1 Hz to 1 MHz. The measurements and computations were carried out automatically. With the commercial interface and automation tool Lab View, data was gathered. For the relative permittivity or the real dielectric constant (ε′) and loss tangent (tanδ), the measurement error percentages are 1 and 3 percent, respectively. Loss tangent is the ratio of loss factor (ε″) to relative permittivity tanδ = ε″/ε′^24^. The samples were investigated at room temperature.

#### Scanning electron microscope (SEM)

The samples microstructure was examined using a scanning electron microscope ‘(SEM) at magnification 7000. The SEM in use is an Energy Dispersive Spectroscopy (EDX) unit +− SEM Philips XL30 Japan model, and it is installed at National Research Centre (NRC).

#### Antimicrobial study

Qualitative evaluations were carried out in nutrient broth according to El-Ansary and El-boraey^21,22^. The pathogenic microorganisms used in this study included Gram-positive bacteria (*Staphylococcus aureus*), Gram-negative bacteria (*Escherichia coli*), and pathogenic yeast (*Candida albicans*). The nutrient broth medium was used to prepare the fresh overnight broth cultures, and qualitative evaluations were conducted in the broth. The cultures were incubated at 37 °C. The inoculum size of this pathogenic strain was prepared and adjusted to approximately 0.5 McFarland standard (1.5 × 10^8^ CFU/ml). 25.0 µL of the inoculum size of each microorganism strain was separately inoculated into each plate containing 20.0 ml of the sterile nutrient agar medium (NA). The prepared non-heat-treated and heat-treated tested cement samples of concentrations (0, 20, and 30%) were divided into two main groups; the first one was applied after the media cooled and solidified on the surface of the inoculated agar plates, which were prepared previously using the disc diffusion method. In this method, each sample was placed separately on the tested strains.

These seeded plates were placed in the refrigerator for one hour for more diffusion of these samples, followed by incubation at 37 °C for 24 h, and zones of inhibition (ZI) were measured in mm.The second group was socked into the NRC tap water separately. By using the shake flask method to calculate the antimicrobial activity, the microbial inhibition was determined by inoculating petri dishes with solidified nutrient agar medium with 100 µL from a (10–4) dilution and calculating the reduction growth rate R(%) for treated samples in relation to control tap water according to the following equation^25^.

$${\text{Relative }}\left[ {{\text{Reduction R}}\left( \% \right)} \right] \, = \, \left( {{\text{A}} - {\text{B}}/{\text{A}}} \right) \, \times { 1}00$$

where A is the number of microorganisms present in the control tap water and B is the number of microorganisms present in tested flasks that contain tap water after applying cement samples.

## Results and discussion

### Green cementanous mortar characterization

This section studies and illustrates the impact of sand substitution with waste from polyvinyl chloride (WPVC) pipes on the physical and mechanical characteristics of cementitious mortars.

#### Dry bulk density

**Figure 2:**
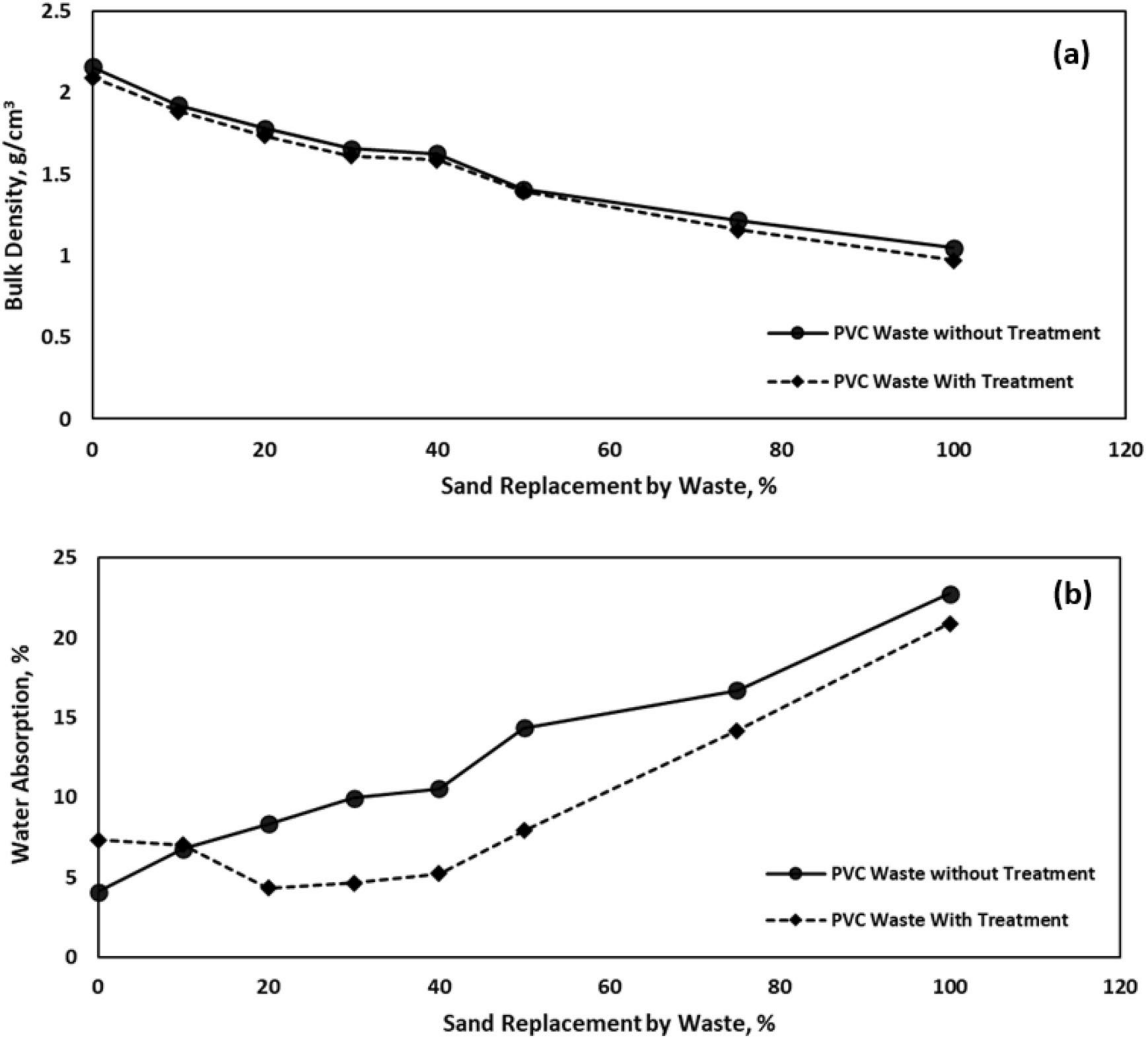
Effect of sand replacement by WPVC wastes on the physical properties of cementitious mortar: (**a**) Dry bulk density; (**b**) Water absorption.

Figure 2a shows the impact of sand substitution by WPVC waste on the dry bulk density of cementitious mortar, before and after heat treatment. This figure shows that the density of cementitious mortars reduces as the amount of waste in the mixture rises. This is so because waste has a significantly lower density than sand. Also, it was evident that the density of the 50% WPVC sample had decreased by roughly 35% when compared to the control sample.

#### Water absorption

The water absorption of cementitious mortar samples was determined. Figure 2b illustrates the effect of sand replacement by WPVC on the percent water absorption where it appears that an increase in waste level causes a corresponding increase in water absorption owing to increased porosity, which is mainly associated with the drop in the bulk density.

#### Mechanical properties

**Figure 3:**
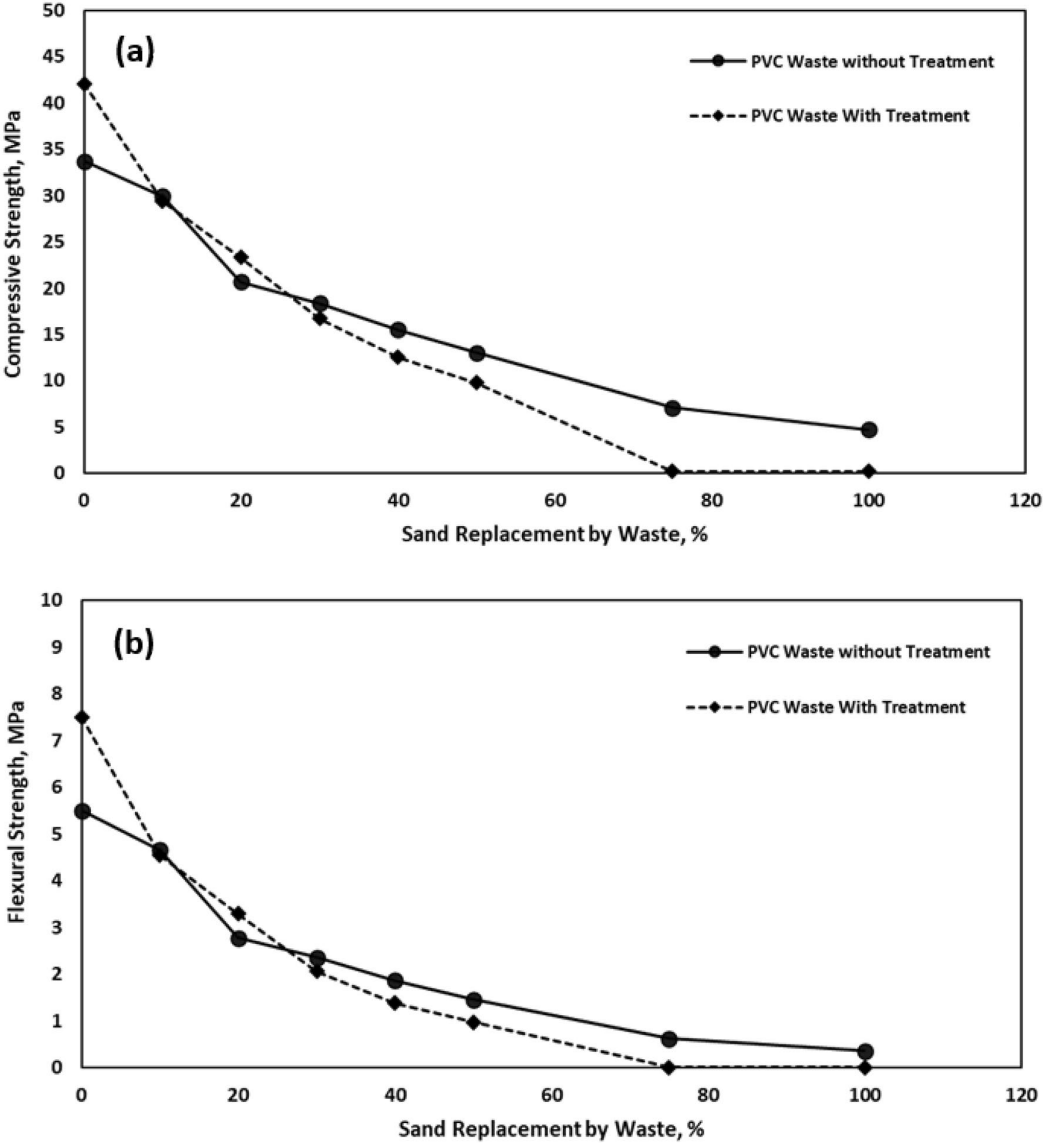
Effect of sand replacement by WPVC on the mechanical properties of cementitious mortar: (**a**) The compressive strength; (**b**) The flexure strength.

Mechanical behaviour of cementitious mortar prepared using WPVC pipes was studied by compression and flexure tests after 28 days of curing. Figure 2a illustrates the effect of sand replacement by WPVC on bulk density, before and after heat treatment. While Fig. 3a,b illustrates the effect of sand replacement by WPVC and heat treatment on the compressive strength and flexure strength of cementitious mortar samples respectively.

It is clear that there was a significant reduction in the mechanical properties (compressive and flexure strengths) of cementitious mortar samples using WPVC after 28 days of curing. The mechanical properties of cementitious mortar samples using WPVC after 28 days of curing, before and after heat treatment, were decreases as the percentage of WPVC waste increases. This pattern may be attributed to the decline in the adhesive strength between the surface of WPVC aggregate and the cement paste, which is due to the difference in particle size, shape and surface texture of the sand and WPVC.

### Quality assessment and compatibility with standards of cement building bricks

From the foregoing discussion, it is apparent that WPVC can be safely incorporated in green cementanous mortar for use in producing cement building bricks. For these products, the cube specimens of (50 × 50 × 50) mm^3^ after 28 days curing were tested. Dry bulk density, water absorption, total linear drying shrinkage, and compressive strength tests, were selected for this purpose. The values obtained for the different properties reported in the American Standards^26^ for cement building bricks are compatible with standard requirements as can be observed from Table 1

**Table 1:** Compatibility of suggested mix with ASTM standard for cement building bricks [ASTM C 55/2017].

Property	Sand replacement	ASTM C 55/2017	
	20% PVC waste	Average of 3 units	Individual units
Visual inspection	All units are sound and free of cracks and other defects	All units shall be sound and free of cracks or other defects that impair the strength or permanence of the construction	
Dry bulk density, kg m^−3^	1780	2000 or more	–
Maximum water absorption, %	4.3	10.4	12
Minimum compressive strength, MPa	23.33	17.2	13.8
Total linear drying shrinkage, %	0.003	Not exceed 0.065%	

### Electrical properties

#### Effect of frequency on the dielectric properties of cement:

**Figure 4:**
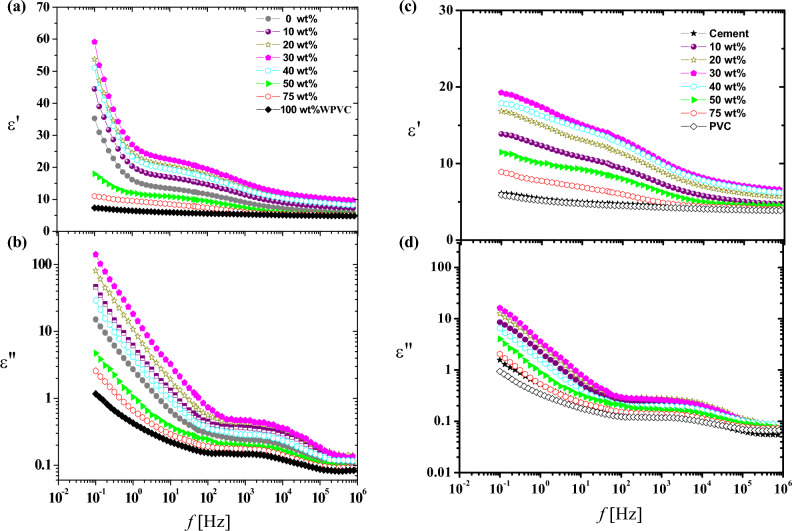
Variation of ε' and ε" versus frequency (**f**) of cement paste with different concentrations of WPVC as a replacement of sand at room temperature: (**a**,**b**) before heat treatment and (**c**,**d**) after heat treatment.

Figure 4 illustrate the dielectric properties of investigated samples. The hardened cement paste has the ability to store energy when it is exposed to an electromagnetic field, so it is considered as dielectric material. So Dielectric relaxation spectroscopy may be considered as non distractive technics for cement paste tasting. The characterization of hardened cement paste can be obtained by investigation of its dielectric properties using electromagnetic (EM) field method^23^. At room temperature, the dielectric properties of cement paste, with different concentrations of WPVC (0, 10, 20, 30, 40, 50, 75 and 100%) as a replacement of sand, were measured as a function of frequency (from 0.1 to 10^6^ Hz). Also the dielectric properties of cement paste were investigated after heat treatment, at room temperature with the same previous concentrations over frequency range (0.1–10^6^ Hz).

##### Before treatment

**Figure 5:**
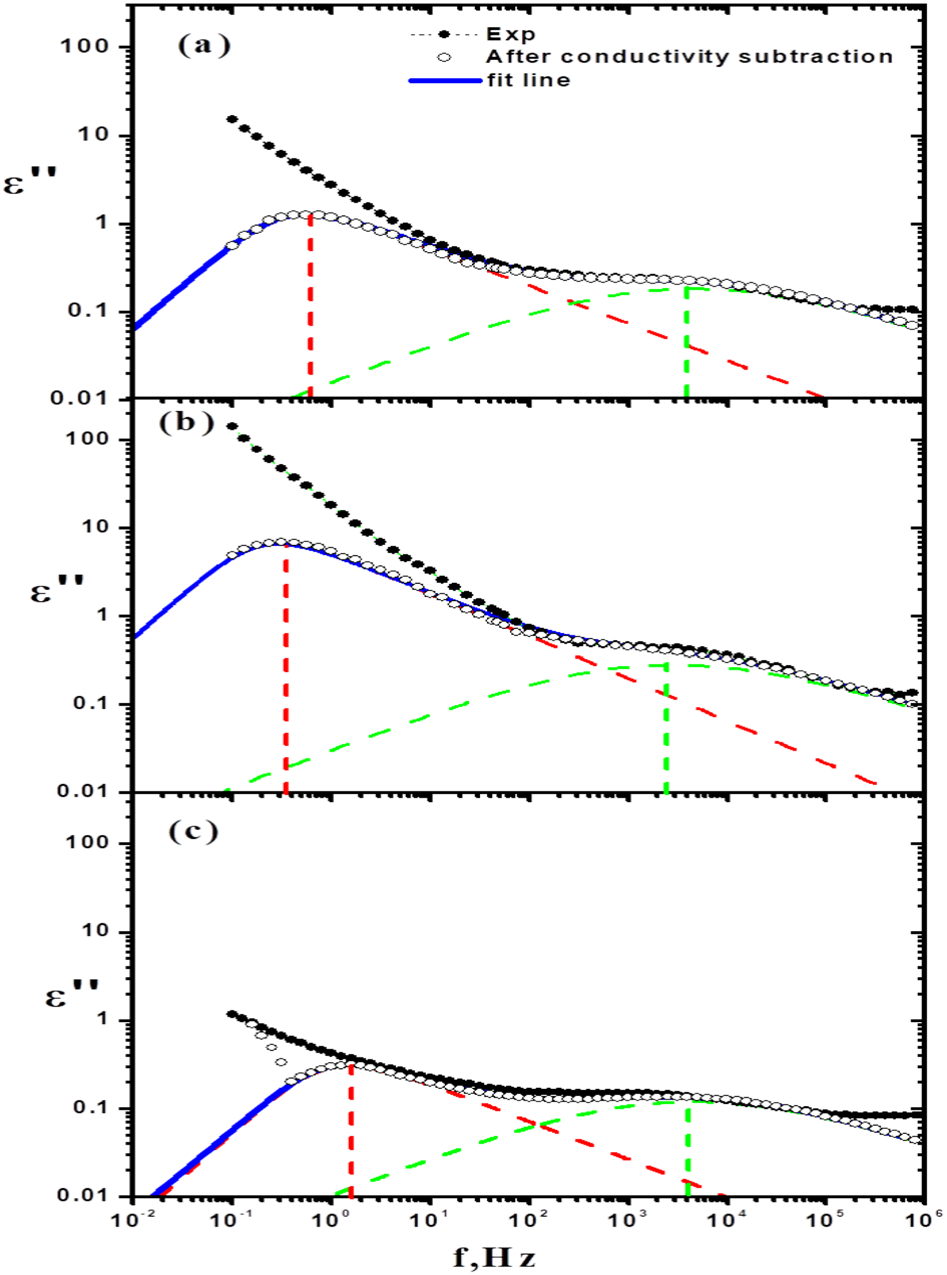
Fitting of dielectric loss ε" data for (**a**) 0%, (**b**) 30% and (**c**) 100% WPVC as a replacement of sand, with Havriliak–Negami functions.

**Figure 6:**
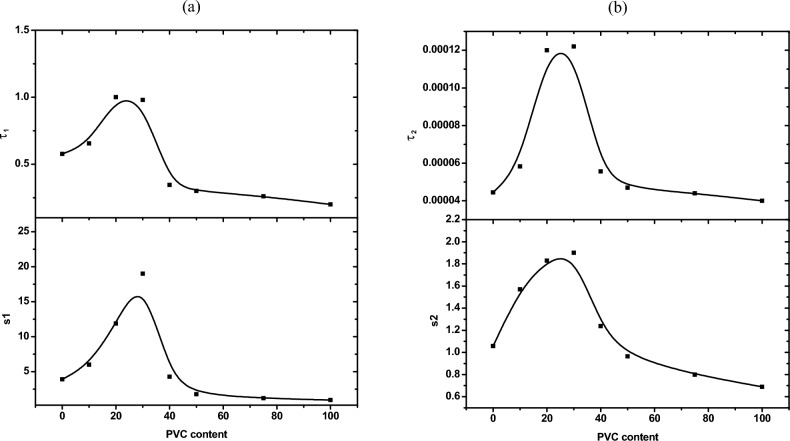
Variation of the relaxation (τ) times and intensities (s) of (**a**) the low frequency process and (**b**) the high frequency process as a function of WPVC content.

**Figure 7:**
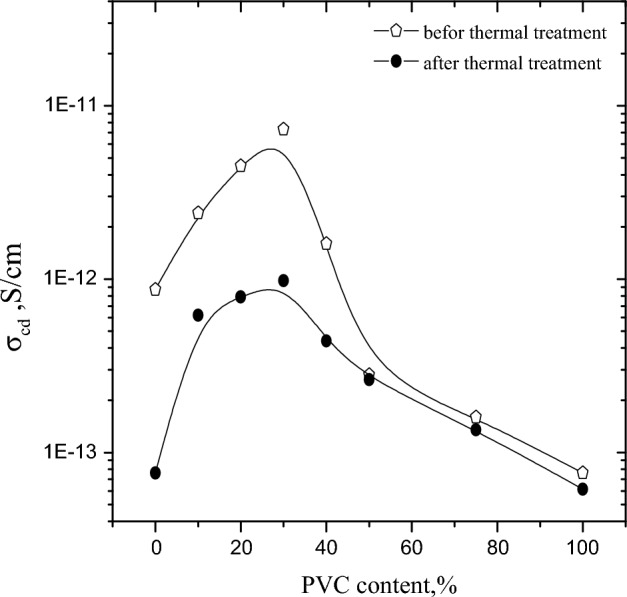
Variation of dc-conductivity versus WPVC content (%) before and after thermal treatment a room temperature.

Figure 4a,b illustrates the dielectric constant (ε′) and dielectric loss (ε″) of cement specimens with (0, 10, 20, 30, 40, 50, 75 and 100% )WPVC respectively .From Fig. 4a, the values of dielectric constant of investigated samples tend to be less with the increasing of frequency, for example decreased from about 60 to about 15 for 30% WPVC. This behaviour is as a result of the polarization phenomenon, polarization phenomenon causes the accumulation of ions and gases at the boundaries of the materials. Figure 4a,b also shows that values of ε′ and ε″ rise as WPVC concentration rises till it reaches 30% (as a replacement of sand). After this concentration, ε′ and ε″ are inversely proportional to WPVC content. We attribute the behaviour of ε′ with addition of WPVC to change in air voids content. Air has the lowest dielectric constant (ε′ = 1(at room temperature), so less air voids content is therefore expected to result in a rise in the dielectric constant^27^. From the information provided above, we may conclude that the increase in dielectric constant up to 30%WPVC is due to a decrease in voids content, which is compatible with SEM images. More addition of WPVC causes formation of its aggregates hence increasing air voids content which is also supported with SEM photos. As a result of the development of charge paths inside the cement paste, a decrease in the air voids content is also expected to raise the dielectric loss, as shown in Fig. 7b. Also, Fig. 4b, shows the trend of dielectric loss with increasing frequency, demonstrates how the dielectric loss reduces with frequency. This may be a result of the phenomenon wherein higher frequency electromagnetic waves are more capable of passing through things without absorbing as much energy^23^. Moreover, Fig. 4b shows that the spectrum of dielectric loss against frequency is broader than the Debye curve, indicating the existence of multiple relaxation processes. A computer program based on Havriliak–Negami function^28^. The best fitting for the data at room temperature was achieved by superimposing two Havriliak-Negami functions in addition to the conductivity function. As an example, Fig. 5a–c illustrates the analysis curves of hardened cement past with concentrations (0, 30 and 100%) of WPVC respectively. The Maxwell–Wagner effect, which is a result of the interfacial polarisation, is responsible for the first relaxation process, which occurs in the low frequency region (at about 1HZ). The interfacial polarization phenomenon arises as a result of a change in the local conductivity across internal interfaces^29^. The several components of the examined systems were responsible for this change in local conductivity. In our research, the occurrence of this phenomenon is thought to be caused by differences in the permittivities and conductivities of the cement paste's ingredients (water, cement, sand, and WPVC).In the hardened cement paste, Water can be in a  bound or unbound chemical state. The unbound chemical state water includes free state and physically bound state. The difference between physically bound water and free water, or normal water, is that in the physically bound water state, the hydrogen bond network between molecules almost has a tetrahedral structure as a result of their molecular contact with other components of cement paste and the electric charge distribution of their electronic orbitals. Hence, bound water exhibits a lower relaxation frequency than free water^30,31^. The second relaxation process lies at high frequency range (about 4 kHz) is attributed to the polarization of physically bound water^29,32^. Figure 6a,b illustrates the relaxation (τ) times and intensity (s) of the two processes as a function of WPVC content respectively.

From this figure, it is clear that the relaxation time and the intensity of the first process (Maxwell–Wagner) increase with WPVC content until (30%). This implies that the density of accumulated charge between interfaces increases with decreasing in their mobility. This may be a result of addition of polar WPVC and voids decrease^31^. Also the relaxation time and the intensity of relaxation process attributed to bound water have the same behavior. The increment in relaxation time, by WPVC addition, means that the bound water is more restricted by stronger hydrogen bonds. The surface of WPVC is polar and hydrophobic due to its high concentration of C–C, C–H, and C–Cl moieties^33,34^. The increase in the strength of the hydrogen bonds with WPVC addition may be due to the fact that the water H bonds in the neighbourhood of totally hydrophobic surfaces is as strong as those in ice or clathrates^35^. On the other hand, the increase in intensity of this process shows more contribution of bound water, which could indicate a rise in the quantity of water molecules that are bound^36^.

##### After treatment

As the cement composites are subjected to long-term temperature, releasing of water occurs basing on energy that binds water to the solid phase. Firstly, free water evaporates above 105 °C, followed by capillary and physically bound water (above120 °C), and finally, water chemically bound in cement hydrates (at very high temperature where cement paste decomposition occurs). Release of bound water has a considerable impact on the mechanical properties of cement pastes, which are 50% dependent on the cohesive forces and bonding given in C–S–H^37^. After exposure of the samples to thermal treatment at t = 250 °C, above WPVC melting point, Fig. 4c,d shows the dielectric constant (ε′) and dielectric loss (ε)″ of all cement specimens after thermal treatment. Comparing Fig. 4a–d, the behaviour is clearly the same after and before heat treatment of cement paste, while the quantity of permittivity (ε′) and dielectric loss (ε″) of cement specimens decreased after heat treatment .This decreasing in (ε′ and ε″) after heat treatment is a result of evaporation of free water^37^, which reduces ion production hence dielectric permittivity^22^. Figure 4a–d illustrates the decreasing ratio in (ε′ and ε″), after heat treatment, against WPVC content. From this figure, it is clear that the decreasing ratio in (ε′ and ε″) decreases as the ratio of WPVC increases, this may be evidence that the amount of free water decreases as the WPVC content, as replacement of sand, increases. This is a logic explanatory because WPVC is a hydrophobic material while sand is a hydrophilic material^38,39^. At high WPVC concentrations, the decrease in free water was expected to enhance the mechanical properties of the cement^33,34^, but the increment of WPVC aggregates, hence increasing air void content, and the increment of pores, confirmed by SEM, prevented this from happening, which agrees with the mechanical results (Figs. 1 and 2).

#### Effect of thermal treatment on conductivity

Variation of dc-conductivity (σ_cd_) versus WPVC content before and after thermal treatment illustrated in Fig. 7. This figure depicts how the dc-conductivity rises with WPVC loading up to 30% concentration. This rising may be attributed to decrement of air voids. After which, σ_cd_ is inversely proportional to WPVC content. The decrease in free water and the increase in air voids are the reasons behind this reflexive behaviour. Also, the figure shows that, before thermal treatment, the dc-conductivity increased from 10^−13^ (for 0% PVC) to10^−12^(until 30%) which lies in antistatic range (10^−12^–10^−9^ S/cm), where the term "anti-static" describes a substance that can be either conductive or dissipative and inhibits the accumulation of static electricity. While the dc-conductivity after thermal treatment increased from 10^−14^ (for treated 0% WPVC) to10^−13^ (for treated 20 and30%) which lies in insulation range (10^−16^–10^−12^ S/cm)^3^, where insulators stop the flow of electric charges, but they do not stop accumulation of static charges.

### SEM Microstructure

**Figure 8:**
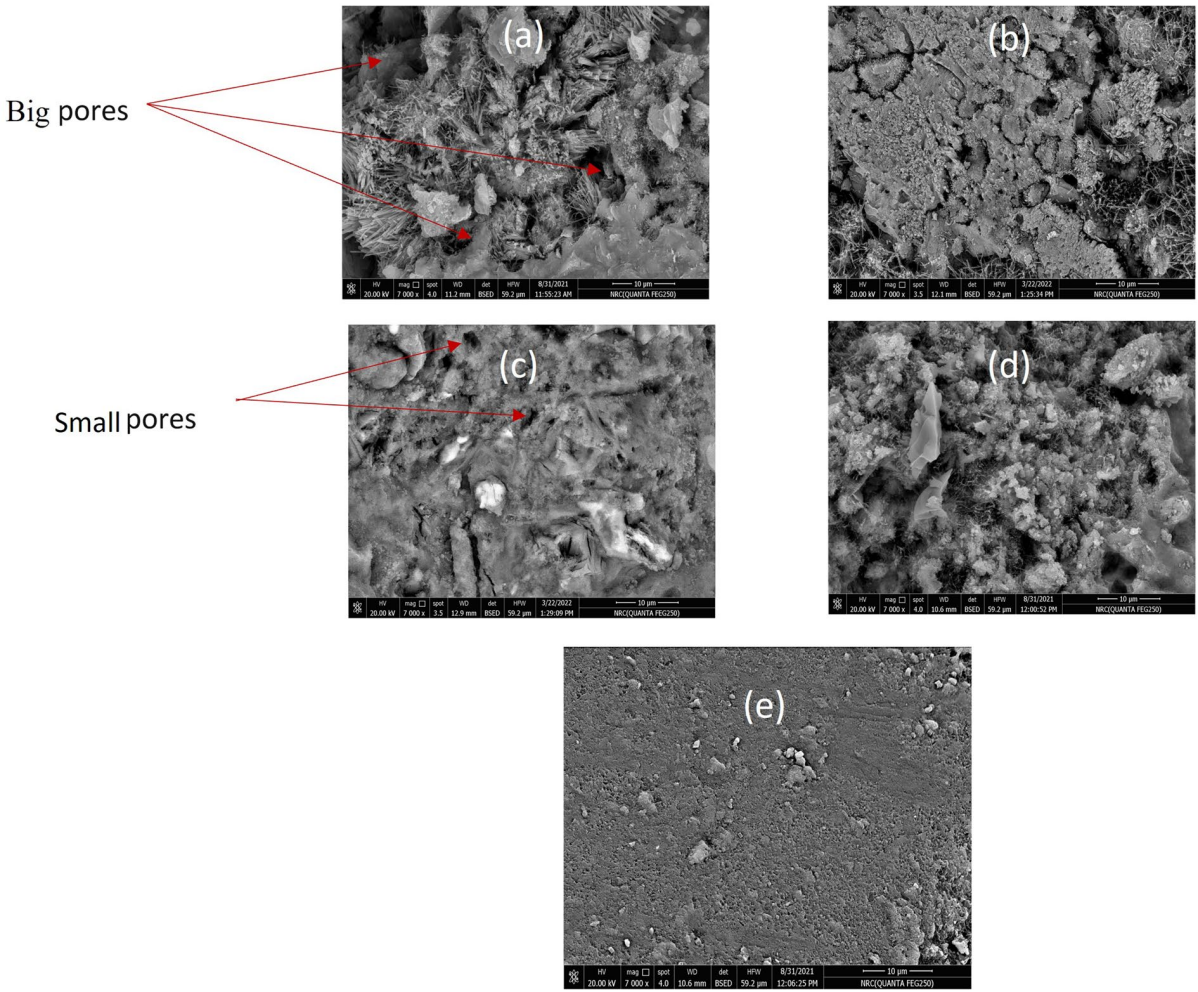
The microstructure of cement specimens with (**a**) 0%WPVC, (**b**) 10% WPVC, (**c**) 20% WPVC, (**d**) 40% WPVC and (**e**) 40% WPVC after heat treatment at about 250 °C.

Figure 8a–d illustrates the microstructure of cement specimens with (0, 10, 20 and 40%) of WPVC respectively. Figure 8a–b illustrates that the number and size of pores/air voids decreases as the concentration of WPVC increases until 20%.

An increase in WPVC content will increase the interaction between its particles. So, the interaction between WPVC and cement paste began to get worse, and the aggregates began to form after a concentration of 30% WPVC^13,40^. Figure 8d illustrates the increase in the number and size of pores/air voids at concentration 40% WPVC. This increase in pores and air voids has effects on the electrical and mechanical properties of cement samples, as described before. Figure 8e illustrates the effect of the temperature, at about 200 °C, on cement specimens with 40% WPVC. By comparing Fig. 8d,e before and after heat treatment, it is clear that the number and size of pores/air voids decrease and the surface of cement specimens is meanly smooth. Smoothing of the surface results from melted WPVC filling the pores and air gaps.

### Antimicrobial studies

**Figure 9:**
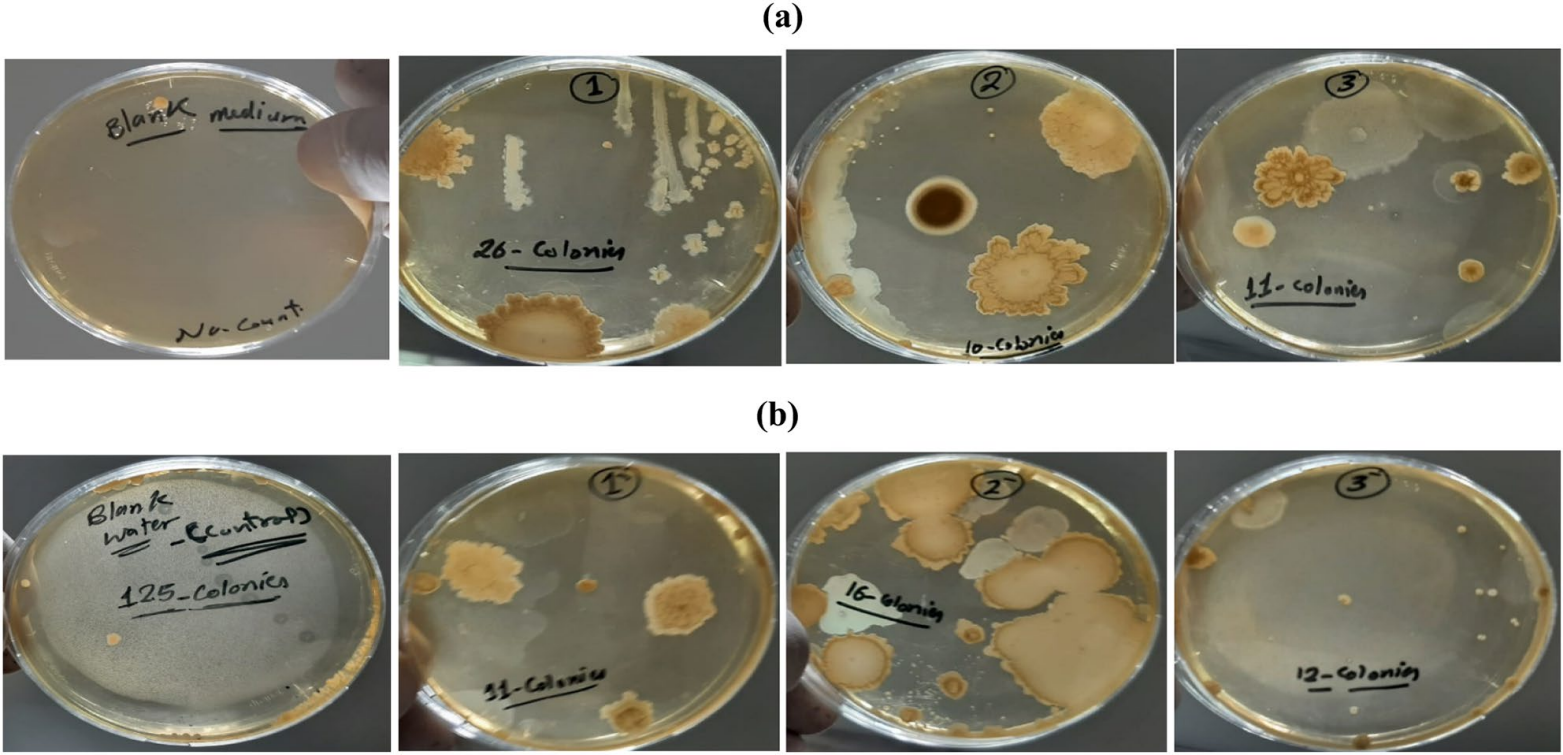
(**a**) Total viable count CFU for non-heat-treated samples (0, 20 and 30%) WPVC and (**b**) heat-treated samples applying plate total viable technique.

**Figure 10:**
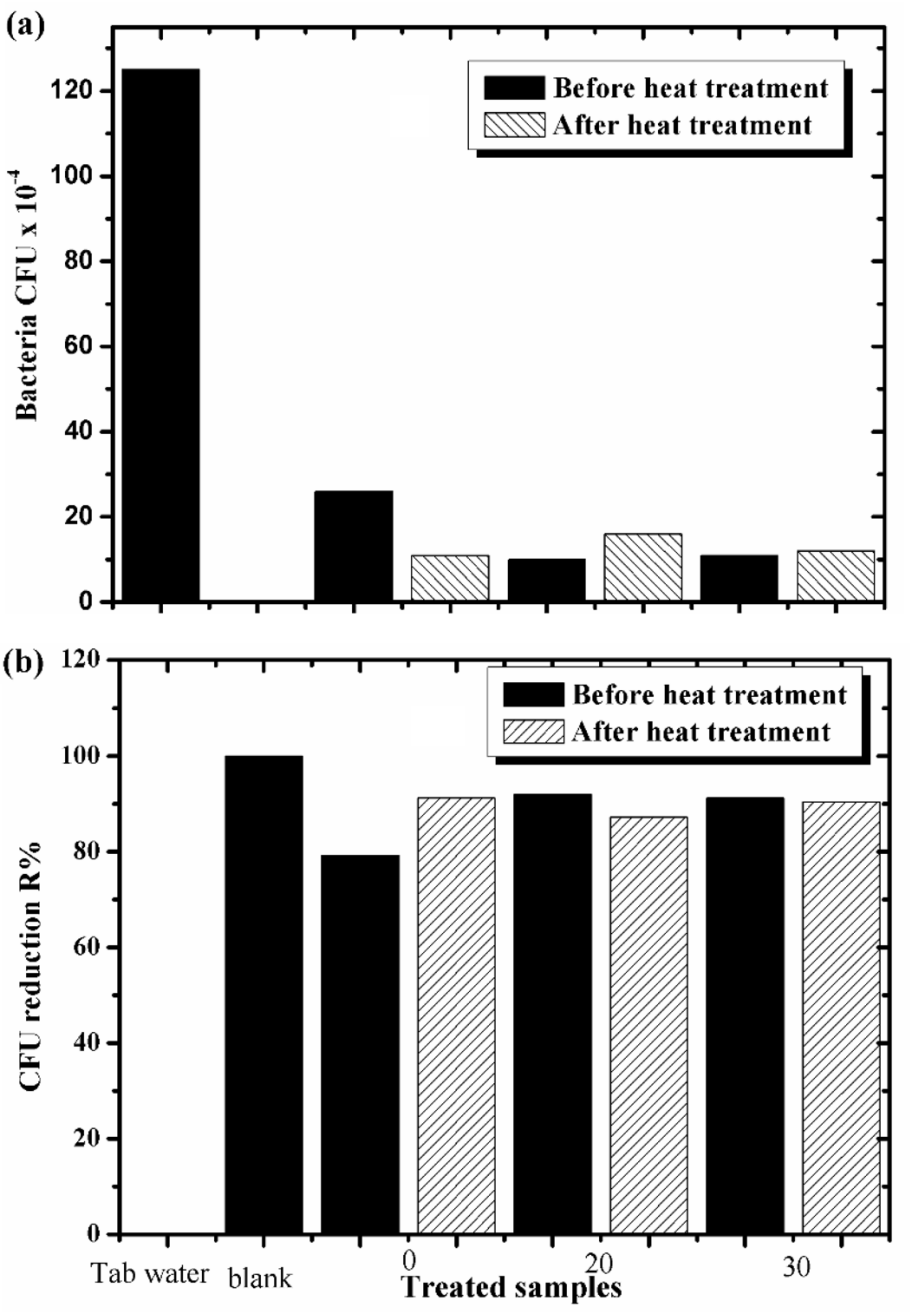
(**a**) Bacterial CFU × 10^−4^ and (**b**) CFU reduction R% heat-treated and non-treated samples of concentrations (0, 20 and 30%) WPVC using shake flask method applying total viable count technique.

The antimicrobial efficacy of the prepared cement samples of WPVC concentrations (0, 20, and 30%) before and after heat treatment was studied against three types of microbes; *Escherichia coli*, *Staphyllococcus aureus*, and the pathogenic yeast Candida albicans. The tested cement samples were divided into two main groups. The first one was studied by applying the disk diffusion method. There is no inhibition zone detected for the prepared samples compared to the control reference medium. Therefore, the antimicrobial efficacy of the cement samples did not appear by this method, so we studied the antimicrobial efficacy by the shake flask method. The second group of the tested samples were soaked in NRC tap water, and the number of microbial colonies CFU formed by the samples and the reduction growth rate R% in relation to control tap water were calculated as described in the experimental part^35^. Figure 9a,b shows the number of colonies in the blank medium which contains the broth medium without any tested sample. The blank medium contains control tap water and the medium contains tap water in which the cement samples were immersed. The calculated data was written in Table 2 and illustrated in Fig. 10a,b. Figure 10a illustrates the relation between the number of grown bacterial colonies (CFU × 10^−4^) and WPVC/cement concentrations. From the graphs, for non-heat-treated samples, we noticed that control tap water, in the absence of cement paste samples, had the highest number of bacterial colonies, which was 125 colonies. When the cement sample of concentration 0% was immersed in water, the number of bacterial colonies was 26; this was approximately reduced by 79.2%. This means the cement paste decreases the number of bacterial colonies that could be grown in tap water. Wherever, the number of grown colonies decreased to 11 colonies, approximately 92%, by WPVC addition to the cement sample of concentration of 30%. The presence of pores and voids enhances bacterial growth, so this decrease in bacterial colonies may be due to a decrease in voids inside the cement sample because WPVC fills the pores. On the other hand, for heat-treated samples, we noticed that the number of grown bacterial colonies for the cement paste of concentration 0% was 11 colonies, which are less than the non-heat-treated sample, which was 26 colonies. This may be due to the evaporation of free water inside its pores, leading to the absorption of some water where the sample was immersed which contains bacterial colonies. So, the actual number of bacterial colonies decreased. Also, we noticed that there is a small difference in the number of microbial colonies in the samples that contain WPVC. This is because WPVC is hydrophobic by nature. So, there is less free water inside the sample. It is a good result that the number of colonies decreased for all the soaked samples compared to control tap water and decreased by introducing WPVC into the cement paste sample. Further, there is a small difference in the number of microbial colonies for treated and non-heat-treated samples that contain WPVC. The relation between CFU reduction R% and WPVC cement concentration was shown in Fig. 10b. We noticed that the value of CFU reduction R% increased for all cement samples. For the non-heat-treated sample of concentration 0%, the CFU reduction R% was 79.2%. This value increased after heat treatment, reaching 91.2%. Also, the CFU reduction R% value increased by the addition of WPVC to the cement paste at concentrations of 20 and 30%, and there was a slight increase for heat-treated samples, as shown in Table 2. So, it was a good result that the antimicrobial properties were enhanced by WPVC addition to the cement paste samples, and all concentrations containing WPVC had the best antimicrobial properties compared to the cement paste samples without it.

**Table 2:** CFU of the tested non-heat-treated and heat-treated samples using shake flask method after incubation applying total viable count technique:

Treated Samples	Bacteria CFU × 10^−4^		CFU reduction R%	
	Before heating	After heating	Before heating	After heating
0	26	11	79.2	91.2
20	10	16	92	87.2
30	11	12	91.2	90.4
Tap water(control)	125		0000	
Blank medium	No count		100

## Conclusion

This study looked at how WPVC affected the microstructure, density, compressive strength, dielectric characteristics, and conductivity of cement paste mixes. WPVC was used in the mix at varying percentages to partially replace fine aggregate. This article also investigated the influence of higher temperature on the compressive strength and dielectric properties of cement pastes which contain WPVC. The following conclusions and recommendations are drawn from the study's findings.

- From SEM photos, the number and size of pores/air voids decrease as the concentrations of WPVC increase until30%, after this concentration ,the number and size of pores/air voids increased with increasing of WPVC ratio. After heat treatment, it is clear that the number and size pores/air voids decrease and the surface of cement specimens is meanly smooth.
- At room temperature, ε′ and ε″ increased with increasing in WPVC percentage until concentration 30%. Due to increase in air avoids content, ε′ and ε″ are inversely proportional to WPVC content after 30%.
- The relaxation time (τ) of the relaxation process attributed to bound water increases with WPVC content until (30%), this implies that the bound water be more restricted, stronger hydrogen bond. Also the intensity (s) of this relaxation process increase with WPVC content until (30%), showing more contribution of bonded water, which may be evidence of an increase in number of bound water molecules.
- After exposure of the samples to thermal treatment at t = 200 °C, above PVC melting point, the decreasing ratio in (ε′ and ε″) decreases as the ratio of WPVC increase, this is may be evidence that the amount of free water decreases as the WPVC content increases.
- Before thermal treatment the dc-conductivity ranged from 10^−13^ to 10^−12^ (until 30%) which lies in antistatic range. After thermal treatment the dc-conductivity ranged from 10^−14^ to 10^−13^ (for 20 and 30%) which lies in insulation range.
- The effect of WPVC addition on free water, bound water and air voids was explained in terms of dielectric behavior. So, Dielectric measurements may be considered as non-destructive testing method for different cement paste mixtures.
- The antimicrobial efficacy of the prepared non heat and heat treated cement samples of WPVC concentrations (0, 20 and 30%) was studied by determining the number of bacterial colony-forming CFU and the reduction growth rate R% when the samples were soacked in tap water. A Cement sample with a concentration of 0 could reduce the number of bacterial colonies in control tab water by 79%. Also, the number of grown colonies decreased by approximately 92% when WPVC was added into the cement sample. It is a good result that the number of colonies decreased for all the samples compared to tab water and decreased by introducing WPVC into the cement paste sample.
- The value of CFU reduction R% increased for all cement samples. For the sample of concentration 0%, this value increased for the non-heat-treated sample. Also, the value increased by the addition of WPVC to the cement paste at concentraeions of WPVC concentration of 20 and 30% compared to that of 0% but the heat-treated samples were affected slightly. So, it is a good result that the antimicrobial properties are enhanced by the addition of WPVC to to the cement paste, and all concentrations of the samples containing WPVC have the best CFU reduction R% compared to the blank cement itself. So, it is recommended to use these samples in places that must be highly clean, such as kitchens, hospitals, and food processing facilities, as the microbial growth will be limited.

## Data Availability

The datasets used and /or analyzed during the current study available from the corresponding author on reasonable request.

## References

[1] Zimbili O, Salim W, Ndambuki M (2014). A review on the usage of ceramic wastes in concrete production. Int. J. Civil Archit. Struct. Construct. Eng..

[2] Amin ShK, Sibak HA, El-Sherbiny SA, Abadir MF (2016). An overview of ceramic wastes management in construction. Int. J. Appl. Eng. Res..

[3] Reffaee AS, El Nashar DE, Abd-El-Messieh SL, Abd-El Nour KN (2009). Electrical and mechanical properties of acrylonitrile rubber and linear low density polyethylene composites in the vicinity of the percolation threshold. Mater. Des..

[4] Safiuddin MD, Jumaat MZ, Salam MA, Islam MS, Hashim R (2010). Utilization of solid wastes in construction materials. Int. J. Phys. Sci..

[5] Xue Y, Hou H, Zhu S, Zha J (2009). Utilization of municipal solid waste incineration ash in stone mastic asphalt mixture: Pavement performance and environmental impact. Construct. Build. Mater..

[6] Aubert JE, Husson B, Sarramone N (2006). Utilization of municipal solid waste incineration (MSWI) fly ash in blended cement, part 1: Processing and characterization of MSWI fly ash. J. Hazard. Mater..

[7] Batayneh M, Marie I, Asi I (2007). Use of selected waste materials in concrete mixes. Waste Manag..

[8] Marzouk OY, Dheilly RM, Queneudec M (2007). Valorization of post-consumer waste plastic in cementitious concrete composites. Waste Manag..

[9] Lei G, Togay O (2016). Use of recycled plastics in concrete: A critical review. Waste Maneg..

[10] Mohammed QF, Zena KA, Suhair KA (2021). A review in sustainable plastic waste in concrete. J. Eng..

[11] Wang YM, Meng YF (2016). Reviewed of antibacterial concrete research and application status. Ningxia Eng. Technol..

[12] Rabin T. & Shi Y. Sustainability of using recycled plastic fiber in concrete. In: *Use of Recycled Plastics in Eco-Efficient Concrete*, 441–460, (Woodhead Publishing, 2019).

[13] Mona S., Abeer R., Shimaa H., Salwa L. A. & Emad S.S. Eco-friendly polymer composite films based on waste polyvinyl chloride/sunflower seed cake for antimicrobial and antistatic applications. Pigment & Resin Technology ahead-of-print (2022).

[14] Hilal NN, Nawar MT, Al-Hadithi AI (2020). Effect of treated polyethylene waste on some mechanical properties of cement mortar. Key Eng. Mater..

[15] Noeiaghaei T, Mukherjee A, Dhami N, Chae S-R (2017). Biogenic deterioration of concrete and its mitigation technologies. Constr. Build. Mater..

[16] Nica D, Davis JL, Kirby L, Zuo G, Roberts DJ (2000). Isolation and characterization of microorganisms involved in the biodeterioration of concrete in sewers. Int. Biodeterior. Biodegrad..

[17] Wei S, Jiang ZL, Liu H, Zhou DS, Sanchez Silva M (2013). Microbiologically induced deterioration of concrete: A review. Braz. J. Microbiol..

[18] Uchida H., Enokida T. & Tanaka R., Tamano M. Deterioration preventive for concrete or mortar and method for preventing deterioration of concrete or mortar. Patent US 6159281 (2000).

[19] Vaquero JM, Cugat V, Segura I, Calvo MA, Aguado A (2016). Development and experimental validation of an overlay mortar with biocide activity. Cem. Concr. Compos..

[20] Xie Y, Lin X, Ji T, Liang Y, Pan W (2019). Comparison of corrosion resistance mechanism between ordinary Portland concrete and alkali-activated concrete subjected to biogenic sulfuric acid attack. Constr. Build. Mater..

[21] Haile T, Nakhla G (2009). Inhibition of microbial concrete corrosion by *Acidithiobacillus*
*thiooxidans* with functionalized zeolite-A coating. Biofouling.

[22] Sihai W, Chung DDL (2001). Effect of admixtures on the dielectric constant of cement paste. Cement Concr. Res..

[23] Jamil M, Hassan MK, Al-Mattarneh HM, Zain MF (2013). Concrete dielectric properties investigation using microwave nondestructive techniques. Mater. Struct..

[24] El-Anssary AA, Abdel Raoof GF, Saleh DO, El-Masry HM (2021). Bioactivities, physicochemical parameters and GC/MS profiling of the fixed oil of Cucumis melo L. seeds: A focus on anti-inflammatory, immunomodulatory, and antimicrobial activities. J. Herbmed. Pharmacol..

[25] Abdelghaffar F, Abdelghaffar RA, Arafa AA, Kamel MM (2018). Functional antibacterial finishing of woolen fabrics using ultrasound technology. Fib. Polym..

[26] ASTM C 55/2017, Standard specification for concrete building brick, ASTM Annual Book, U.S.A., 04 (June 2018).

[27] Elboraey AN, Abo-Almaged HH, El-Ashmawy AAER, Abdou AR, Moussa AR, Emara LH, El-Masry HM, El Bassyouni GET, Ramzy MI (2021). Biological and mechanical properties of denture base material as a vehicle for novel hydroxyapatite nanoparticles loaded with drug. Adv. Pharm. Bull..

[28] Hammami H, Arous M, Lagache M, Kallel A (2007). Study of the interfacial MWS relaxation by dielectric spectroscopy in unidirectional PZT fibres/epoxy resin composites. J. Alloys Compd..

[29] Samet M (2015). Electrode polarization versus Maxwell–Wagner-–Sillars interfacial polarization in dielectric spectra of materials: Characteristic frequencies and scaling laws. J. Chem. Phys..

[30] Kaatze U (2011). Bound water: Evidence from and implications for the dielectric properties of aqueous solutions. J. Mol. Liq..

[31] Robin kent, in energy manegment inplastic processing (third Edition) (2018).

[32] Ashraf S (2013). Compressive strength and electrical properties of cement paste utilizing waste polyethylene terephthalate bottles. J. Appl. Sci. Res..

[33] Ahmad A, Márian L, Petr S, Miran M (2012). Recent progress in surface modification of polyvinyl chloride. Materials.

[34] Matej K, Alexander S, Emanuel S, Roland RN (2016). Water-mediated interactions between hydrophilic and hydrophobic surfaces. Langmuir.

[35] Grdadolnik J, Franci M, Franc A (2017). Origin of hydrophobicity and enhanced water hydrogen bond strength near purely hydrophobic solutes. Proc. Natl. Acad. Sci..

[36] Keskin SO, Sumnu G, Sahin S (2007). A study on the effects of different gums on dielectric properties and quality of breads baked in infrared-microwave combination oven. Eur. Food Res. Technol..

[37] Prochon P, Piotrowski T (2016). Bound water content measurement in cement pastes by stoichiometric and gravimetric analyses. J. Build. Chem..

[38] Chen L (2017). Superhydrophobic sand: A hope for desert water storage and transportation projects. J. Mater. Chem. A.

[39] Hilal NN, Mohammed TN, Abdulkader IA (2020). Effect of treated polyethylene waste on some mechanical properties of cement mortar. Key Eng. Mater..

[40] Roshanaei H, Khodkar F, Alimardani M (2020). Contribution of filler–filler interaction and filler aspect ratio in rubber reinforcement by silica and mica. Iran. Polym. J..

